# Mortality and Medical Care after Bereavement: A General Practice Cohort Study

**DOI:** 10.1371/journal.pone.0052561

**Published:** 2013-01-25

**Authors:** Michael King, Mira Vasanthan, Irene Petersen, Louise Jones, Louise Marston, Irwin Nazareth

**Affiliations:** 1 Unit of Mental Health Sciences, Faculty of Brain Sciences, University College London Medical School, London, United Kingdom; 2 Research Department of Primary Care and Population Sciences, University College London Medical School, London, United Kingdom; Cardiff University, United Kingdom

## Abstract

Bereaved spouses or partners are thought to be at increased risk of morbidity and mortality. However, there are few large prospective studies and results are inconsistent. We estimated the relative mortality, prescription of psychotropic medication and use of primary medical care services in adults whose cohabitee died of cancer. To do this, we undertook a cohort study using The Health Improvement Network (THIN) UK primary care database. Participants were 1) people aged over 40, who were registered with general practices and had been exposed to the death of a cohabitee from cancer; and 2) a comparison cohort frequency matched on five year age bands and sex who were cohabiting with a living partner. The baseline was chosen as six months before the date of the cancer death for the exposed group and a random date for the unexposed group. Incidence rate ratios (IRR) with 95% confidence intervals (CI) were estimated using random effects Poisson regression to account for clustering within general practices and adjusting for other key variables. 92,129 patients were studied for a median follow up of 4 years. Cohabitees of patients who died of cancer were less likely to die of any cause (IRR 0.71, CI 0.68–0.74) after adjustment for age, gender, number of non-psychotropic prescriptions 6 months before the cancer death/index date, use of psychotropic medication 6 months before the cancer death/index date, smoking, alcohol and area deprivation score. Exposed patients were more likely to receive a prescription for antidepressant or hypnotic medication and to attend their GP both before and after the death of the cohabitee. In conclusion, we did not confirm increased mortality in cohabitees of people dying from cancer.

## Introduction

Grief is the constellation of psychological and physical reactions to the death of a spouse, relative, child or friend [1]–[3]. Bereavement is a universal experience and is viewed by the public as the most stressful of all life events [3]. It has long been recognised that it may place older spouses and partners at risk of adverse outcomes including increased morbidity and mortality [2]. Possible reasons for elevated mortality have included emotional stress and its somatic consequences, shared environmental risk factors such as smoking or diet, increased use of alcohol and recreational drugs, and poor self and/or health care following bereavement. However, there are few large scale prospective studies of the health outcome of spouses and partners bereaved by cancer and results are inconsistent.

Grief is a complex process. The early effects for a person who loses a partner or spouse concern a) the immediate shock and psychological impact of bereavement and b) the change from cohabitee/married to single status [4]. Although depression or other psychological distress may diminish over the first year, distress over the loss itself may continue for a number of years [5] and include deterioration in morale and in physical health. In studying the impact of bereavement on longer term physical health it is important to distinguish any such changes from normal age related deterioration, and thus controlled comparisons are essential. The clearest evidence for increased mortality arises from a study of members of a health care plan in California in which 12,522 spouse pairs were followed up for 23 years. Mortality following bereavement was significantly higher in men and women after adjusting for age, education, and other predictors of mortality. The highest relative risks occurred 7–12 months after bereavement but remained elevated for two years after the bereavement [6]. The study, however, was unable to adjust for other important changes in health status in the “to-be bereaved” or those that might have occurred between the baseline examination and mortality, possibly weakening the relationship between risk factors other than bereavement and mortality. Other studies have reported absent or statistically weaker associations. A large cohort study of 7,735 men followed for a mean of 11.5 years in the United Kingdom showed no association between becoming widowed and subsequent cardiovascular or non-cardiovascular (usually cancer) death [7]. This was in contrast to men divorced during follow-up who were at elevated risk of both types of mortality. A further study of data on almost 92,000 people in the US identified 4,032 white adults who became widowed between 1963 and 1974. It was reported that mortality rates based on person-years at risk were not elevated for widows compared to matched controls remaining married, while the relative risk was significant for men in only those 55 years old and over [8]. Another large US study of mortality over 11 years estimated that the relative risk of mortality was greater for unmarried versus married partners from all ethnic backgrounds [9]. Unfortunately this study did not compare mortality in all bereaved participants versus those not bereaved.

The mechanisms involved in men's particular vulnerability are not clear short of the somewhat prosaic suggestions that men are less able to prepare nutritious meals and are more likely to smoke and misuse alcohol. In order to explore whether sex differences in such factors after bereavement might explain higher risk in widowers than widows, data were examined from a national register in Denmark of over 6,000 bereavements [10]. Although daily use of medication and primary care visits rose for up to five years after bereavement, no differences between men and women were found. However, a number of other studies have reported age and gender differences in vulnerability to bereavement [11]–[13].

Given these varying results, we undertook a study of bereavement using The Health Improvement Network (THIN), a national primary care database in the UK. Our key objectives were to estimate 1) the mortality, 2) prescription of psychotropic medication and 3) use of general practice services in adults whose cohabitee died of cancer and compare them to a cohort of individuals of similar age and gender who continued to cohabit with another adult. We selected one particular type of illness (cancer) to reduce some of the variability inherent in the data. These are patients and families who receive many similar types of treatment support and for whom the death is expected to some degree.

## Methods

### Ethics statement

The THIN scheme for obtaining and providing anonymous patient data to researchers was approved by the National Health Service South-East Multicenter Research Ethics Committee (MREC) in 2002.

### Data source

The Health Improvement Network (THIN) is a primary care database of anonymous current and historical general practice records from 1987 to the present day on more than 9 million patients from nearly 500 practices in the UK (www.cegedimstrategicdata.com). THIN is a well validated and widely used source of real time clinical data which has extensively been used for research purposes [14], [15]. The database is based on practices which use the Vision software to record patient care and management. It includes information on patient demographic details [date of birth, gender and social deprivation (quintiles of Townsend deprivation scores)] as well as registration details. Longitudinal data on diagnoses, symptoms, procedures, investigations and additional health information such as smoking and substance use is largely recorded using Read codes a hierarchical coding system [16], together with data on all prescriptions issued (see below). Drugs prescribed are mapped to chapters in the British National Formulary, so it is possible for researchers to select all (or some if more appropriate) drugs from a given chapter. Each patient also has a family number, which identifies people who are living in the same household or otherwise are associated. The database is broadly representative of UK general practices in terms of patients' age and sex, practice size and geographical distribution [17]. Over 98% of the UK population is registered with a general practitioner [18].

### Practices and participants

As some practices did not use their computer systems fully in the early 1990s we used data from practices from the time point where they met our predefined criteria for acceptable computer usage: that on average registered patients have at least one medical record, one additional health record and at least two prescriptions recorded per year [14], [19]. Furthermore, it was a requirement that practices recorded death as expected for the practice's age and gender distribution [20]. Exposed individuals were patients aged over 40 who lived with someone who had died from cancer when they were also aged over 40. Relatively few people die of cancer below the age of 40, we therefore decided to focus our study on those above that age. We avoided the death of children in families by restricting the age gap between participant and bereaved to no more than 15 years. Exposed patients were identified by first extracting cancer deaths and subsequently identifying individuals with the same family number registered with the general practice at least six months before the death of the deceased. We restricted the sample to those with at least 6 months data in order to have sufficient data before the death against which to measure change. We also restricted our sample to households with two adults with less than a 15 year age gap between them, and up to four children under the age of 18 years. This was to exclude instances where the family number represents blocks of flats or residential homes.

We identified a comparison cohort of up to five unexposed for each exposed individual using the same criteria except that their cohabitee was still alive at a randomly chosen index date and throughout the follow-up period. We needed to select a start point for data collection for the unexposed group. However, given there was no obvious date for the unexposed, we selected a random date within the time period where they were registered with the GP, the logic being that had their partner died it would have been on a random date during their registration. The random date was allocated by selecting time from a uniform distribution after the time when data were of sufficient quality and the potential unexposed patient and partner fulfilled the age criteria. We stratified the sample in terms of sex and age (within five years age bands) to ensure similar distributions amongst the two groups and thereby gain greater statistical power. Patients were followed up until they died, left the practice or end of data collection.

### Exposure

The exposure was bereavement due to a cancer death.

### Outcomes

Outcomes were mortality, courses of medication to alleviate psychological distress and GP consultations after the cancer death/index date.

### Potential confounders

The following variables were included in the analyses as potential confounders: age (five year age bands), gender, number of general medicine prescriptions six months before the cancer death/index date, and whether or not psychotropic medication was prescribed [antidepressants, antipsychotics, hypnotics (yes/no)] in the six months before the death. All prescribing is now done through the practices' computer systems and the classes of prescribed drugs are arranged according to the chapters of the UK's British National Formulary. This includes obsolete medications, so patients prescribed drugs that are no longer possible to prescribe are included in the data. Patients were classified as current smokers or non-current smokers (to include ex-smokers). Patients with missing smoking data (4%) were classified as non-current smokers. Excessive alcohol consumption was classified as percentage of women who consumed over 14 units of alcohol per week, and men who consumed over 21 units per week. We assumed everyone else had within guidelines drinking habits, which gave rise to no missing data. These data were taken from a recording at any time, with the closest to the baseline being used. These data are collected by the general practice staff at a new patient health check on registration or if the information is relevant to the disease for which the patient has consulted (e.g. if a patient presents with respiratory symptoms, smoking status may be enquired about). Other data may be part of the UK's Quality Outcomes Framework [21] for people with a number of chronic conditions. These people are more likely to have such data recorded than those without these conditions. For example if you are a smoker you will be asked on regular interval whether you are still smoking. However, this is not the case if you are a non-smoker. In short, the vast majority of patients have at least one measurement of these health indicators during the time they are registered with a GP.

### Statistical Analysis

The main outcomes were first examined using descriptive statistics. We used the Kaplan-Meier estimator to estimate the survival function of the exposed and unexposed cohort and crude rates of mortality were compared. Crude mortality rates were expressed as number of deaths per 1000 person years. Unadjusted and adjusted incidence rate ratios (IRR) with 95% confidence intervals were estimated for the association between bereavement and the main outcomes. We used Poisson regression as the cohorts in this study are dynamic i.e. patients register and leave the practices at different time points. Random effect Poisson regression also takes account of practice clustering. We tested formally whether the effect of bereavement differed by age using a log likelihood ratio test in the adjusted model. This determined whether there was an interaction between age (dichotomised as under or over 60) and whether the study population was part of the exposed (to cancer death) or unexposed. Finally, we explored whether mortality varied within the exposed group by gender. The analysis was conducted using Stata 11.

### Missing data

In clinical practice, patients often have measurements of blood pressure, weight and height, alcohol consumption and smoking status taken when they are relevant to current or future health status. However, we found in another study that over two thirds have these measurements taken in the first year after they register with a general practice [22]. After the introduction of the Quality Outcomes Framework the level of recording of these health indicators has increased in particular for those who suffer from chronic conditions included in that Framework. However, few subjects in this study had missing data (e.g. only 4% had no information available on smoking). For medical conditions and prescriptions we assumed that if there was no record, the patient had not had it within the relevant time window.

## Results

### Baseline characteristics

In total, there were 15,748 exposed individuals living with a person diagnosed with cancer 6 months before their death and 76,381 unexposed individuals with an adult cohabitee at a random index date. We cannot know how many participants were excluded on the basis that they were potentially residing in flats or residential homes as that criterion was a part of the extraction algorithm – they were not all extracted first and then those in group residences excluded. Table 1 shows the demographics and other characteristics of the exposed and unexposed cohorts. The first entry into the cohorts was in 1987 and the last exit was in 2010. Median duration of follow-up was four years. Mean ages were identical in the exposed and unexposed groups (71, SD 10 years), while 10,308 (65%) of the exposed and 45,593 (60%) of the unexposed were women. Ninety-eight per cent of exposed and unexposed households contained opposite sex dyads. Twenty-eight per cent of exposed households were in areas with a deprivation score of 1 (least deprived) compared to 31% of the unexposed. More of the exposed than unexposed households (10% vs. 7%) were in the most deprived area. Prevalence of smoking was higher in the exposed than unexposed group [3,099 (20%) vs. 12,012 (16%)] while excessive alcohol consumption was less frequent in the exposed cohort [2,273 (14%) vs. 11,918 (16%)].

**Table 1:** Description of exposed and unexposed cohorts.

	Exposed	Unexposed
Number of participants	15,748	76,381
Mean age (SD)	71 (10)	71 (10)
Women	10,308 (65%)	45,593 (60%)
Median (25thquartile, 75^th^ quartile) follow-up time years	4.24 (1.81, 7.45)	3.99 (1.78,6.77)
Male/female pairs	15490 (98%)	74730 (98%)
Psychotropic medication Prescriptions issued 6 months before cancer death date/index date^1^		
Antipsychotic N (%)	207 (1%)	866 (1%)
Hypnotic N (%)	1,503 (10%)	4.379 (6%)
Antidepressant N (%)	1,983 (13%)	7,361 (10%)
Other prescriptions issued Median (25^th^ percentile, 75^th^ percentile) number of prescriptions issued 6 months before cancer death date/index date	3 (1,6)	3 (1,6)
Use of substances^2^		
Current smokers	3,099 (20%)	12,012 (16%)
Excessive alcohol use^3^	2,273 (14%)	11,918 (16%)
Townsend Deprivation Score in quintiles: N (%)^4^		
1 lowest deprivation	4,362 (28%)	23.724 (31%)
2	3.841 (24%)	19.694 (26%)
3	3.086 (20%)	14,768 (19%)
4	2.574 (16%)	10,671 (14%)
5 highest deprivation	1.552 (10%)	5,486 (7%)

1 Expressed as the number who ever had a prescription over the 6 months.

2 All available records of subjects were searched for information on substance use.

3 Percentage of women who consumed >14 units of alcohol/week, and men who consumed >21 units/week.

4 2% of the exposed and 3% of the unexposed did not have data on deprivation.

### Findings for the 6 months before cancer death/index date

In total, 12,756 (81%) of the exposed and 62,632 (82%) of the unexposed received at least one prescription for a non-psychotropic medication in the six months before the cancer death/index date. The median numbers of prescriptions were also similar in both groups [exposed group median 3 (25^th^ and 75^th^ centiles 1, 6) and unexposed group 3 (1, 6)]. However, prescriptions for psychotropic medications were higher in the exposed group [hypnotics 1,503 (10%) vs. 4,379 (6%); antidepressants 1,983 (13%) vs. 7,361 (10%); and antipsychotic prescriptions 207 (1.3%) vs. 866 (1.1%)]. Table 2 shows Incidence Rate Ratios (IRR) for hypnotic, antidepressant and antipsychotic use, and GP consultations in the exposed and unexposed cohorts six months before the cancer death/index date. Prescriptions for hypnotic (IRR 1.59, 95% CI 1.49–1.69) and antidepressant use (IRR 1.26, 1.20–1.33) were significantly higher in the exposed group after adjustment but there was no significant difference in antipsychotic use (IRR 1.09, 0.93–1.27). GP consultations were higher in the exposed compared to the unexposed group (IRR 1.21, 1.20–1.22).

**Table 2:** Prescription of psychotropic medication and consultations with GP over 6 months before cancer death/index date.

Baseline	Unadjusted IRR (95%CI)*	Adjusted IRR (95%CI)* #
Hypnotic use	1.69 (1.59–1.79)	1.59 (1.49–1.69)
Antidepressant use	1.33 (1.27–1.40)	1.26 (1.20–1.33)
Antipsychotic use	1.16 (0.99–1.35)	1.09 (0.93–1.27)
Consultations with GP	1.21 (1.20–1.22)	1.18 (1.17–1.18)

* Reference group unexposed.

# Adjusted for age, gender, number of non-psychotropic prescriptions, smoking, alcohol and deprivation score.

### Psychotropic prescriptions and primary care consultations after the death/index date

Patients exposed to the cancer death were significantly more likely than comparison patients to receive new prescriptions for hypnotics (IRR 2.44, 1.89–3.14) and antidepressants (IRR 1.87, 1.53–2.29) (Table 3). However, there was no significant difference in new antipsychotic prescriptions (IRR 0.95, 0.49–1.84). Exposed patients consulted their GPs slightly more frequently than unexposed during follow-up (IRR 1.06, 1.06–1.07) (Table 3)

**Table 3:** Mortality, incident# prescriptions for psychotropic medication and consultations with GP after cancer death/index date.

Outcome	Unadjusted IRR(95%CI)*	Adjusted IRR(95%CI)*
All cause mortality	0.70 (0.67–0.73)	0.71 (0.68–0.74)^1^
Incident hypnotic prescription	2.52 (1.95–3.24)	2.44 (1.89–3.14)^2^
Incident antidepressant prescription	2.02 (1.66–2.47)	1.87 (1.53–2.29)^3^
Incident anti psychotic prescription	1.02 (0.53–1.97)	0.95 (0.49–1.84)^4^
Consultation with GP	1.08 (1.08–1.09)	1.06 (1.06–1.07)^5^

# new courses, not prescribed in the 6 months before the death/index date.

* Reference group unexposed.

1 5 Adjusted for age, gender, number of non-psychotropic prescriptions 6 months prior to cancer death/index date, use of psychotropic medication 6 months prior to cancer death/index date, smoking, alcohol and area deprivation score.

2 3 4 Adjusted for age, gender number of non-psychotropic prescriptions 6 months prior to cancer death/index date, smoking, alcohol and area deprivation score.

### Mortality

**Figure 1:**
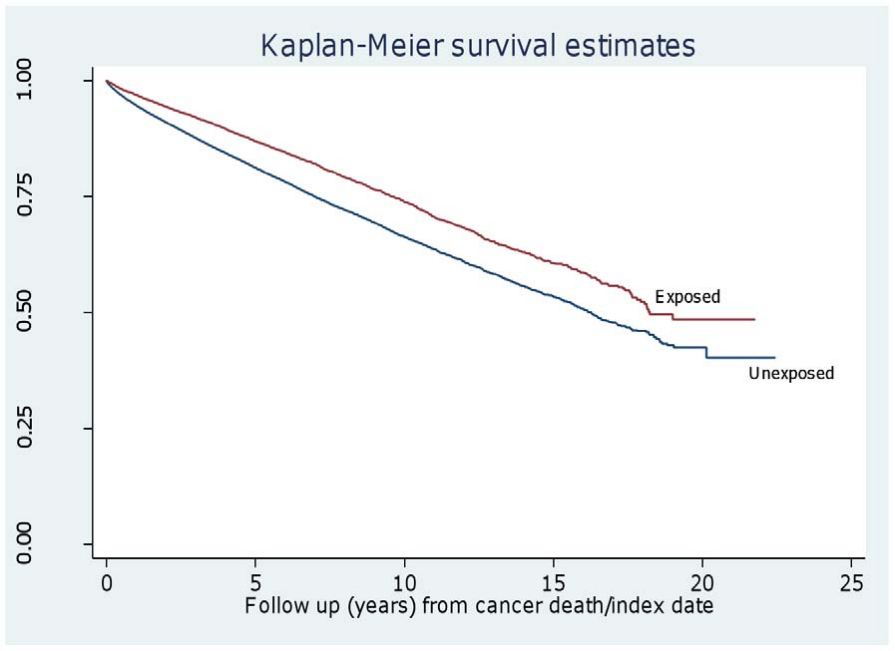
Kaplan-Meier survival probabilities in exposed and unexposed groups after cancer death/index date.

All cause mortality was lower in the individuals whose spouse died from cancer (exposed) (29.96 per 1000 person years) than in those in the unexposed group (42.29 per 1000 person years) (Figure 1) and this remained after adjustment (IRR 0.71, 0.68–0.74). The effect of bereavement was the same across ages, in that there was no interaction between age (over and below the age of 60 years) and exposure groups (p = 0.28).

### Sex differences in mortality rates in the exposed cohort

Except for the youngest age group (40–49 years), men had consistently higher mortality than women in each age group and this became more marked with increasing age (Table 4).

**Table 4:** Crude mortality rates within 10 year age bands for the exposed cohort stratified by gender after cancer death.

Age band	Men (Rate/1000 person years)	Women (Rate/1000 person years)
40–49	4.42 (2.11–9.27)	2.95 (1.41–6.18)
50–59	9.82 (7.14–13.49)	4.99 (3.57–6.98)
60–69	20.74 (17.87–24.08)	13.71 (12.13–15.50)
70–79	49.60 (45.28–54.33)	29.19 (26.95–31.61)
80 and over	117.26 (106.20–129.48)	76.59 (69.82–84.01)

## Discussion

We found that individuals exposed to a cancer death had a reduced risk of mortality 2) an increased likelihood of receiving a new prescription for an antidepressant or hypnotic; and 3) a higher consultation rate with the general practitioners during a median of four years after the death of their partner. Psychotropic medication was also more commonly prescribed to exposed participants six months before the death strongly suggesting they were already requiring help to cope with a stressful situation as their cohabitee approached death. Our finding of a reduced mortality after the death of a spouse, friend, partner or sibling runs in contrast to some previous research. However, in this study we focused on bereavement following a cancer death whereas previous studies have not made this distinction.

Our finding of increased survival of a spouse or partner after a cancer death is not completely counter-intuitive. The release from the stress of caring may lead to better health and reduced psychological morbidity for partners as the acute phase of grief subsides. Furthermore, one possible explanation for our finding is that many patients dying of cancer in the UK receive greater supportive and palliative care services than those dying from other causes and this may moderate cohabitees' extreme stress [23] [24]. Where specialist rather than generalist palliative care is available, carers may receive more direct support. Many community and hospice based specialist palliative care teams conduct bereavement risk assessments in relatives and friends of the dying [25], [26]. Pre-bereavement formal and informal counselling may be available for families, and many palliative care services get back in touch with families in the first few weeks of bereavement to offer further support. This is particularly common practice in hospice settings, and the majority of deaths in hospices are still due to cancer. NICE guidelines in England and Wales for specialist palliative care recommend carers are fully involved in clinical decisions and are supported emotionally [27]. Death is also a more expected outcome following a diagnosis of cancer. Those who have been diagnosed early may have had considerable time to prepare for death. People with cancer are more likely to be placed on registers as part of the Gold Standards Framework, a structured care programme in UK general practice http://www.goldstandardsframework.org.uk/), and be offered opportunities to discuss future care planning and preferred priorities for care and place of death. Furthermore, cancer may be distinct from other causes of mortality in that life habits (except possibly smoking) might not be shared between partners as is often the case for COPD, and heart and liver disease. However, even if we are incorrect in this suggestion, one of the best longitudinal studies of the impact of bereavement found little evidence for impact of a shared environment [6]. There are also opportunities for surviving partners to assess and tackle their own health risks during the time from cancer diagnosis to death. Although there is increasing emphasis on good generalist and specialist palliative care for those with advanced progressive illness without a cancer diagnosis [28], many difficulties remain in making this possible. Our findings suggest there may be benefits for carers as well as cancer patients in pursuing the further development of such care for those in the last year of life and those close to them.

General practitioners may play an important part in reducing mortality after bereavement. In the 1980s it was reported that 76% of widowed people had at least one consultation with their family doctor in the five to seven months after the death, despite it being uncommon for bereaved patients to have any record of the bereavement in their GP case-notes [29]. Such attendance figures are likely to be even higher today [30] as it appears that most bereaved patients welcome the involvement of their family doctor [31]. The GP may have had an even greater impact on our exposed group as our study design means that each dyad was registered with the same general practice. When the same GP has been managing cancer in the lost loved one, he or she is more likely to have also helped the bereaved. This is borne out by our results on use of primary care services and is supported by other evidence that relatives of patients who receive terminal care at the same practice fare best, with most being visited by the GP over the year after the death [32]. Our finding of an increase in receipt of prescriptions for antidepressants and, in particular, hypnotics, both before the death of the cohabitee and thereafter, confirm that this is a very stressful time for people and that they suffer in particular from insomnia. Unfortunately, there is little evidence for effectiveness of hypnotics in this setting [33].

Our study has a number of strengths and limitations. This study is based on a very large cohort of people bereaved after the death of a person with cancer and not from couples volunteering for a study of bereavement. They were matched on age and sex with an even larger cohort of controls and data were available for 6 months before the death/index date. Thus the sample was not affected by selection, recall and non-response biases that have limited many studies of bereavement. Use of Poisson regression models, with our main outcomes modelled as time-dependent, allowed for unbiased estimates of the effects of bereavement while reducing bias caused by differential lengths of follow-up. General practice may be an important confounder in that is associated with the exposure and the outcomes. Thus, we used a random effects approach, which took account of the potential clustering effects of general practices. Our main limitation is selection of participant dyads. We cannot be certain that every participant selected was in a coupled relationship (partners or spouses) with each cohabitee; some may have been blood relatives or friends. However, the disparity of sex in 98% of the pairs strongly suggests that the overwhelming majority were cohabiting couples. Furthermore, this limitation can be applied equally to both cohorts. We may also have missed couples living with offspring older than 18 years. However, once again this would have affected both cohorts. The apparent psychological stress reflected in the increased prescriptions also gives grounds for confidence that we have selected couples where the death of the cohabitee had a major impact suggesting that most were emotionally close. We shall also not have captured bereaved people not registered with general practitioners. This limitation, however, is likely to be very small, given that on average 98% of people in England are registered with a general practitioner and that older people, and particularly those with a sick person in the household, are more likely to be registered. Bereaved people who leave their practice (and thus no longer contributed to follow-up data) might be a group at higher risk of death, for example they may include people who can no longer care for themselves and are admitted to residential homes, or move to join their families. We could not trace death certification in people who left the practices as the THIN data are anonymised. To a certain extent, however, this may also have been the case for older people who left the comparison cohort. Although there were some differences between the exposed and unexposed cohorts at the baseline, adjustments in the statistical model would have taken these into account. At first sight, 15,748 cancer deaths is lower than expected for a total practice population of close to nine million patients. The main reason why we have a smaller sample of deaths is because our entry criterion required that the deceased have lived with another adult. Many of those who die from cancer will be living alone as they are widowed or single and do not live with other family members. The other reason is that our cohort is dynamic, so even though there are nine million patients not all were a part of it for the full 15 years. Finally, there may be some cancer deaths that we did not capture as we could not identify the cause of death.

The main focus of the research was on the period after the death (or index date in the comparison patients). The 6 months before the death/index date was chosen as a consistent baseline against which we could measure change. In order to consider data on covariates, a period is needed in which patients are registered with GP. Although an alternative approach would have been to start each cancer dyad at the time of the cancer diagnosis, we would have had a greatly variable time period before the death (in some cases more than a decade), which would not always have been informative since there are many hidden biases (e.g. socio-economic status) for when patients are diagnosed. The same partner might not have been with the deceased throughout this time. Finally it would have made the selection of a start point for control patients inconsistent.

In conclusion, although bereavement led to a higher rate of prescription of psychotropic medication and GP attendances, people living with a partner who dies of cancer appear to have a reduced risk of all cause mortality. Primary care practitioners need to be aware of the increased consultations with cohabiters of people who have recently died from cancer even if the risk of death in the cohabiters is reduced. The tendency to use psychotropic medication such as antidepressant and hypnotic soon after the death of a person with cancer needs further research evaluation.
